# Estimating the cost-effectiveness of the Sodium Reduction in Communities Program

**DOI:** 10.1017/S1368980021004419

**Published:** 2022-04

**Authors:** Benjamin Yarnoff, Emily Teachout, Kara MacLeod, John Whitehill, Julia Jordan, Zohra Tayebali, Laurel Bates

**Affiliations:** 1 RTI International, Research Triangle Park, NC 27709, USA; 2 Centers for Disease Control and Prevention, Atlanta, GA, USA; 3 Deloitte Consulting, LLP, London, UK; 4 IHRC, Inc., Atlanta, GA, USA

**Keywords:** Sodium, Cost-effectiveness, Community health interventions

## Abstract

**Objective:**

This study assessed the cost-effectiveness of the Centers for Disease Control and Prevention’s (CDC’s) Sodium Reduction in Communities Program (SRCP).

**Design:**

We collected implementation costs and performance measure indicators from SRCP recipients and their partner food service organisations. We estimated the cost per person and per food service organisation reached and the cost per menu item impacted. We estimated the short-term effectiveness of SRCP in reducing sodium consumption and used it as an input in the Prevention Impact Simulation Model to project the long-term impact on medical cost savings and quality-adjusted life-years gained due to a reduction in CVD and estimate the cost-effectiveness of SRCP if sustained through 2025 and 2040.

**Setting:**

CDC funded eight recipients as part of the 2016–2021 round of the SRCP to work with food service organisations in eight settings to increase the availability and purchase of lower-sodium food options.

**Participants:**

Eight SRCP recipients and twenty of their partners.

**Results:**

At the recipient level, average cost per person reached was $10, and average cost per food service organisation reached was $42 917. At the food service organisation level, median monthly cost per food item impacted by recipe modification or product substitution was $684. Cost-effectiveness analyses showed that, if sustained, the programme is cost saving (i.e. the reduction in medical costs is greater than the implementation costs) in the target population by $1·82 through 2025 and $2·09 through 2040.

**Conclusions:**

By providing evidence of the cost-effectiveness of a real-world sodium reduction initiative, this study can help inform decisions by public health organisations about related CVD prevention interventions.

High sodium intake can lead to hypertension and increase the risk for heart disease and stroke^(1,2)^. In 2014, US adults between the ages of 20 and 69 consumed sodium at an average of 3608 mg/d^(3)^. The *2020–2025 Dietary Guidelines for Americans* ^(4)^ and the Dietary Reference Intakes for Sodium and Potassium^(5)^ recommend adults consume no more than 2300 mg/d of sodium each day. It has been estimated that every 1000 mg/d increase from this recommendation increases the risk of CVD events by 17 %^(2)^. A large proportion of the sodium consumed in the USA comes from processed foods and foods prepared in restaurants and cafeteria settings – sources over which consumers have little control^(6)^. Accordingly, this gap between recommended intake and actual intake among US adults requires a public health approach that expands beyond a focus on individual behaviour change. Public health approaches to sodium reduction should include strategies that focus on reducing the sodium content in prepackaged and pre-prepared foods^(7,8)^.

The Sodium Reduction in Communities Program (SRCP) began as a demonstration project in the USA in 2010 to address this growing public health concern. As part of the 2016–2021 round of SRCP, the Centers for Disease Control and Prevention funded eight recipients, including local and state health departments and a research university, to work with food service organisations in eight settings to increase the availability and purchase of lower-sodium food options, with the goal of reducing sodium intake to within the recommendation of the *2015–2020 Dietary Guidelines for Americans*. The programme focuses on four distinct sodium strategies: (1) implementation of food service guidelines and nutritional standards that include sodium; (2) introduction of meal and menu item modifications; (3) integration of lower-sodium food procurement practices and (4) implementation of behavioural economic strategies to promote lower-sodium items (e.g. placement interventions).

Early outcome data suggest that partnering with local food service organisations to provide consumers with lower-sodium options is an effective strategy to lower population-level sodium consumption^(9)^. However, little is known about the cost-effectiveness of strategies implemented in SRCP, which is important for public health policy and planning decisions. In this study, we aimed to provide this information by estimating the cost of achieving implementation outcomes and the cost-effectiveness of strategies implemented in SRCP. First, we estimated the cost per unit of improvement in implementation outcomes, such as persons reached, and food items affected by sodium reduction efforts. This approach provides evidence of the cost of achieving implementation objectives. Second, we estimated the long-term cost-effectiveness of SRCP by integrating the estimates of short-term implementation outcomes and costs with a simulation model, the Prevention Intervention Simulation Model (PRISM)^(10–12)^. These estimates of cost-effectiveness can be used to support decision-making about future sodium reduction efforts.

## Methods

### Programme description

Eight recipients were funded as part of the 2016–2021 round of SRCP. Funded recipients include state (New York and Oregon) and local (Los Angeles County, Marion County-Indiana, New York City, Seattle and King County, and Philadelphia) public health departments and a research university (University of Arkansas for Medical Sciences). The recipients partnered with food service organisations to implement sodium reduction strategies in ≥ 1 of eight settings: worksites, hospitals, schools, early childhood education centres, higher-learning institutions, restaurants, emergency food services and distributive or congregate meal sites. Recipients identified and recruited food service organisation partners based on their target populations and partner openness to implementing sodium reduction strategies.

Recipients and their partner food service organisations worked to implement the four strategies of SRCP. Table 1 shows examples of implementation activities for each sodium reduction strategy. Table 2 summarises implementation in each of the eight settings including the number of recipients working in each setting, the number of food service organisations reached in each setting and the number of people reached in each setting. The populations reached in each setting differ most notably in the frequency with which they are reached. For example, schools, early childhood education centres and distributive/congregate meal programmes reach their populations regularly (e.g. schools provide lunch to students every day). Conversely, restaurants may reach different people each day.

**Table 1:** Example implementation activities for each sodium reduction strategy

Sodium reduction strategy	Example activities
Implementation of food service guidelines and nutritional standards that include sodium	• Using environmental scans to inform recommendations for sodium reduction improvements in the food service organisations
	• Convening meetings with key partners like registered dietitians and food service personnel to provide unified guidance for nutrition guidelines and other sodium reduction strategies
	• Setting feasible sodium reduction goals by targeting the food categories that offer the greatest opportunity for reducing sodium (e.g. high sodium ingredients that affect many menu items)
Introduction of meal and menu item modifications	• Developing a culinary food preparation programme where an external chef educated food service staff on culinary techniques and food preparation practices to reduce sodium in meals
	• Distributing lower-sodium cooking materials including recipes, tool kits and measuring spoons to enable chefs to make lower-sodium meals from scratch
	• Conducting skills-based training to enable food service staff to feel more confident using lower-sodium and healthier alternatives while cooking
Integration of lower-sodium food procurement practices	• Embedding sodium standards within existing and new food services contracts for cafeterias
	• Reviewing product lists and provided food service organisations with a list of similar products with lower-sodium content
	• Developing new procurement relationships with vendors and manufacturers who carry lower-sodium food items
Implementation of behavioural economic strategies to promote lower-sodium items	• Redesigning spaces by repainting and hanging photos of healthy foods
	• Discounting purchases that are part of healthy foods programme
	• Removing salt packets from trays and salt shakers from tables so that patrons must request them

**Table 2:** Summary of implementation in each setting, 1 September 2016–31 December 2018

Settings	Number of recipients working in the setting	Number of food service organisations reached	Number of people reached*
Worksites	1	5	16 000
Hospitals	2	19	2 246 000
Schools	3	218	626 000
Early childhood education centres	1	57	5000
Higher-learning institutions	3	22	373 000
Restaurants	1	15	5000
Emergency food services	1	5	228 000
Distributive/congregate meals	5	188	403 000
Total	17	529	3 902 000

* Estimated based on the average number of people served each day in participating food service organisations. Rounded to the nearest thousand.

### Data collection

#### Cost data

We used two different data collection tools to collect data on SRCP implementation costs. The first collected implementation costs from recipients and the second from partner food service organisations. We collected data on implementation costs from recipients using an Excel-based cost-collection instrument. The instrument used an activity-based costing approach^(13)^. Respondents were asked to report all resources (labour and non-labour) used to implement SRCP for six categories: (1) labour; (2) materials, travel and equipment; (3) contracted services; (4) indirect and overhead costs; (5) in-kind labour and (6) in-kind non-labour. In-kind costs included costs incurred to support programme implementation but not paid for using funds from the cooperative agreement such as staff time paid for by the health department and resources donated by the health department. Within each resource category, respondents were asked to allocate each line item across five main programme activities: (1) building and maintaining partnerships, (2) designing sodium reduction interventions, (3) implementing sodium reduction interventions, (4) performing administrative activities and (5) conducting evaluations. We collected data from the recipients in February 2018 to report all costs incurred to implement the programme from 30 September 2016 through 31 December 2017 (15 months); in February 2019 to report all costs incurred from 1 January 2018 through 31 December 2018 (12 months); and in December 2019 to report all costs incurred from 1 January 2019 through 29 September 2019 (9 months). The funded costs and in-kind costs were similar for the first two reporting periods and lower for the third. Combined, these costs represent implementation costs for the first 36 months of the programme. Data were reported by SRCP programme managers with input from other programme staff for all eight recipients. We provided technical assistance for data collection by answering respondents’ questions via email and phone over the data collection period and conducted a data quality review upon submission.

During May–June 2019, we collected data on the in-kind contributions of partner food service organisations to SRCP implementation over the period 30 September 2016 through 30 April 30 2019, using a cost survey. All partner costs were considered in-kind because they were paid for by the partner organisations themselves. In the partner food service organisation cost survey, respondents were asked about a set of key sodium reduction activities including implementation of nutrition guidelines, recipe development and modification, changing food procurement practices, modification to food preparation practices, healthy food promotion, meetings and other activities. For each activity, respondents were asked the number and types of staff who worked on the activity, the average monthly number of hours each staff member worked on that activity and the number of months worked by each staff member. Respondents reported the monthly average across the reporting period. Additionally, for each activity, respondents were asked to report any non-labour expenditures like materials and supplies. Participation was voluntary. Recipients provided contact information for forty-five of eighty-eight key partner food service organisations. We sent invitations to those forty-five food service organisations and received completed surveys from twenty (44 %). The only information we had about partner characteristics was the venue in which they worked. We examined the completion rate for each venue to assess potential response bias: six out of eight congregate meal partners, one out of one early childhood education centres, zero out of three emergency food services, three out of ten higher learning institutions, two out of eight hospitals, two out of two restaurants, four out of ten schools and one out of three worksites.

#### Programme implementation and effectiveness data

As part of SRCP, recipients conduct programme evaluations including the collection and reporting implementation and short-term effectiveness performance measures at baseline (2015–2016) and each programme year thereafter (2016–2017, 2017–2018 and 2018–2019). Centers for Disease Control and Prevention offered a list of implementation and effectiveness outcomes that the programme, as a whole, aims to achieve and ways to measure the performance towards those outcomes. Centers for Disease Control and Prevention provided guidance on data sources and method for computing each performance measure and recipients followed these approaches. Recipients selected target performance measures that fit their needs and capacity levels. Because all recipients did not select the same performance measures to report, data are not available on all measures for all venues. Recipients were encouraged to collect and report performance measure data for all venues in which they worked but some did not collect all measures in all venues. Therefore, performance measures are not representative of all recipient activities. Some recipients reported performance measures even more finely, down to the specific partner food service organisation. For recipient-level analysis, we aggregated partner-level data for each recipient to create a recipient average. For partner-level analysis, we utilised these finer data to link partner performance measure data with partner cost data (*n* 13).

We used four measures of programme implementation for the analysis of the cost of achieving implementation outcomes in the present study: (1) number of food service organisations reached, defined as the number of food service organisations that partnered with the recipient to implement sodium reduction strategies (reported by recipients for seventeen out of the seventeen venues); (2) number of people reached per day, defined as the average number of people served by partner food service organisations each day computed from sales data provided by food service organizations (reported by recipients for seventeen out of the seventeen venues); (3) number of menu items affected by recipe modification, defined as the number of menu items served by partner food service organisations for which the recipe was modified to reduce sodium computed from recipe data provided by food service organisations (reported by recipients for twelve out of the seventeen venues) and (4) number of menu items affected by procurement changes to substitute ingredients or entire items, defined as the number of menu items served by partner food service organisations that were replaced with a lower-sodium alternative entirely or in part (i.e. one ingredient) through changes in procurement computed from procurement records and menus provided by food service organisations (reported by recipients for fourteen out of the seventeen venues). We also used two short-term programme effectiveness measures as inputs in the analysis of long-term cost-effectiveness in the present study: (1) change in average daily sodium intake as measured by the average sodium content of purchased food, computed from a combination of sales data and menu nutrition data provided by food service organisations (reported by recipients for six out of the seventeen venues); and (2) percentage of people in the targeted food service organisation who purchased lower-sodium items computed from sales data provided by food service organisations (assumed to be the percentage of people reducing sodium consumption) (reported by recipients for fourteen out of the seventeen venues).

### Data analysis

We assessed (1) the cost of achieving implementation outcomes and (2) the potential long-term cost-effectiveness of sodium reduction strategies.

#### Analysis of the cost of achieving implementation outcomes

We assessed the costs of a one-unit increase in implementation outcome measures (e.g. person reached) by mapping expenditures reported in the cost study with implementation outcome measures reported by recipients in their performance reporting. We conducted implementation outcome analyses separately for recipients and partner food service organisations that participated in the cost study.

We computed the total cost of all activities and average cost of each activity across recipients. We aggregated total costs across recipients to compute total programme costs. We subtracted the total evaluation costs, as they were not intended to contribute to implementation. We aggregated the number of food service organisations reached and the number of people reached annually in implementation. We then combined cost and reach to estimate the recipient cost per food service organisation reached and per person reached as
(1)

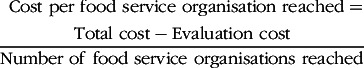



(2)

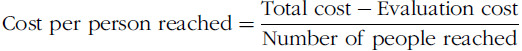

These two metrics represent key implementation outcomes for recipients. Their primary goal is to recruit food service organisations to implement sodium reduction strategies and then catalyse change in those organisations to reach people with sodium reduction strategies.

We computed the average cost per food service organisation to implement each activity. Not all food service organisations engaged in all activities, so not all incurred costs related to each activity. We also computed total cost per person served to account for differences in size across food services organisations. For a subset of thirteen food service organisations, recipients reported organisation-specific data for the performance measure: number of items with lowered sodium through recipe modification or item or ingredient substitution. For this subset of food service organisations, we linked the performance measure data with the cost data and computed cost per food item affected as
(3)

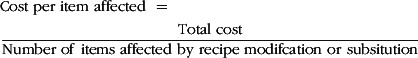

The primary goal of food service organisations was to reduce sodium content of menu items, so this metric represents a summary of their activities. However, it is possible that total cost includes some costs related to activities not specifically aimed at reducing sodium content of food items (e.g. administrative meetings).

#### Long-term cost-effectiveness analysis

We used PRISM to simulate the potential long-term health outcomes and medical costs if reductions in sodium consumption are sustained. This modelling process included five steps: (1) generating estimates of the short-term programme effectiveness on reduced sodium consumption to be used as a model input, (2) estimating the long-term health gains and medical cost savings from sustained reduction in sodium consumption from SRCP, (3) estimating the long-term costs of sustaining sodium reduction strategies, (4) computing the cost-effectiveness ratio and (5) conducting sensitivity analysis of key assumptions.

*(1) Generate an estimate of short-term programme effectiveness*

The PRISM module for examining the impact of changes in average sodium consumption requires, as an input, the population-level reduction in average sodium consumption achieved across recipients which is computed as
(4)

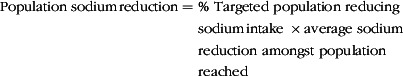

It is important to include the percentage of people reducing sodium consumption as an input, because PRISM is a population model and models the impact across the entire target population. We took the values to compute this input from two short-term programme effectiveness performance measures reported by SRCP recipients: (1) percentage of people in the targeted food service organisation who purchased lower-sodium items (assumed to be the percentage of people reducing sodium consumption) and (2) change in average daily sodium intake as measured by nutritional analysis of items purchased at participating food service organisations conducted by the recipient. As noted above, the performance measure for the percentage of people reducing sodium consumption is based on sales data of the percentage of people that purchase lower-sodium menu options. The food service organisations participating in SRCP serve largely the same customers every day, so this is a reasonable proxy.

The average percentage of people in the targeted population that purchased lower-sodium items was 20 % across all reporting SRCP venues (ranging from 1 to 91 % across venues), and the average reduction in sodium intake was 399 mg per person across all reporting SRCP venues (ranging from 1 to 542 mg across venues). Using these inputs in Equation (4) generates the PRISM input for the short-term effectiveness of the programme as 79 mg/d reduction in sodium consumption across the target population (i.e. 20 %*399 mg/d).

*(2) Estimate the long-term health gains and medical cost savings from SRCP*

We used the estimate of the short-term effectiveness of SRCP as an input in PRISM to produce estimates of the impact of SRCP on per capita health and economic outcomes through 2025 and 2040, including quality-adjusted life-years (QALY), premature deaths and medical costs (medical expenditures). PRISM simulates the relationships between risk factors (e.g. high sodium intake), chronic disease (e.g. hypertension) and health outcomes (e.g. CVD events, deaths and medical costs) annually and cumulatively through 2025 and 2040. Because the model has been described in detail elsewhere^(10–12,14)^, we only focus on the aspects related to sodium in this paper. The model tracks average daily sodium consumption at the population level for a nationally representative population over time and simulates the impact of changes in sodium consumption on hypertension rates in the population. The model then simulates the impact of hypertension on CVD and medical costs, including costs related to hypertension management, CVD and event hospitalisation and care. Costs are all discounted by 3 % annually to account for time preference (i.e. that the present is valued more than the future). Key model parameters related to hypertension and sodium are shown in Table 3. The impact of hypertension on CVD events is modelled using a modified version of the Framingham equation^(15)^. The original Framingham equation was modified for PRISM to (1) include additional risk factors such as secondhand smoke, fruit and vegetable intake, sodium intake, psychological distress and physical activity; (2) include risk adjustments for control of high blood pressure, high blood cholesterol and diabetes; (3) calibrate the CVD event and death rates by age, sex and event type (stroke, CHD and overall) to reported surveillance data; and (4) differentiate rates for first-time and subsequent CVD events. The model simulates changes in risk factors and outcomes over time with and without any intervention and compares the scenarios to estimate the impact of the intervention. PRISM has been validated over the course of its development^(16)^ and has been used to estimate the potential long-term impact and cost-effectiveness of several other community prevention programmes, such as the Communities Putting Prevention to Work programme^(17,18)^ and the Community Transformation Grants programme^(19)^. Specific to the present paper, PRISM includes the ability to model interventions for reducing average sodium consumption in the population.

**Table 3:** Key PRISM parameters related to sodium consumption, hypertension and CVD

Model parameter	Value	Source
Reduction in SBP among the population without hypertension per 1000 mg sodium reduction	1·0 mm Hg	Synthesis of Midgley *et al*.^(20)^, Cutler *et al*.^(21)^, He and MacGregor^(22,23)^, Yang *et al*.^(24)^, He *et al.* ^(25)^, Aburto *et al*.^(26)^, Shi *et al*.^(27)^, Caldiera *et al*.^(28)^
Reduction in SBP among the population with hypertension per 1000 mg sodium reduction	2·75 mm Hg	Synthesis of Midgley *et al*.^(20)^, Cutler *et al*.^(21)^, He and MacGregor^(22,23)^, Yang *et al*.^(24)^, He *et al*.^(25)^, Aburto *et al*.^(26)^, Shi *et al*.^(27)^, Caldiera *et al*.^(28)^
Reduction in CVD events* and deaths from changes in hypertension	Framingham equation	Anderson *et al*.^(15)^
Annual per person cost of hypertension management (2018 $)	$564	MEPS 2003–05 (regression analysis)
Annual acute care and rehab costs of a survived CVD event* (2018 $)	$32 640	Russell *et al*.^(29)^, Carlson *et al*.^(30)^, expert opinion of stroke subject matter experts at Veterans Health Administration, Fox *et al*.^(31)^
Annual acute care costs of a non-sudden death from a CVD event* (2018 $)	$34 643	Russell *et al*.^(29)^
Annual acute care costs of a sudden death from a CVD event* (2018 $)	$1409	Russell *et al*.^(29)^
Average hospitalisation cost of non-CVD† complications of hypertension (2018 $)	$11 910	Average hospitalisation cost of hypertension: HCUP, 2016. ICD-10: I10 (primary hypertension) and I12 (hypertensive CKD)

SBP, systolic blood pressure; PRISM, Prevention Impacts Simulation Model; CKD, chronic kidney disease; HCUP, Healthcare Cost and Utilization Project; ICD-10, International Classification of Diseases, 10th Revision; MEPS, Medical Expenditure Panel Survey.

* CVD events include CHD, heart failure and stroke.

† Non-CVD complications of hypertension include primary hypertension and kidney disease.

*(3) Estimate the long-term costs of sustaining sodium reduction strategies*

PRISM uses the costs per capita for start-up and for ongoing maintenance as inputs. The costs measured in the cost study represent per capita implementation costs for the first 36 months of the programme from recipients and food service organisations. We assumed that this represents the start-up period of the programme. We also assumed that the average cost per capita for the included food service organisations is representative of food service organisations across the programme. Because we only collected implementation cost data during the start-up period, we assumed that the ongoing maintenance costs would be 95 % of start-up costs. This is based on subject matter expert opinion that policy and systems interventions such as these have minimal ongoing maintenance costs, 10 % of start-up (M. Farrelly, personal communication, June 2012). Annual start-up costs used in the model were $2·02 per capita, including costs of recipients and food service organisations. Ongoing maintenance costs were $0·20.

*(4) Compute the cost-effectiveness ratio*

To assess the long-term cost-effectiveness, we computed
(5)

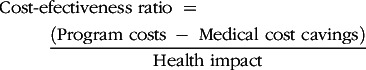

This ratio represents the cost per health impact achieved. It can be thought of as measuring the programme’s return on investment. We measured health impact in terms of both premature deaths averted and QALY gained. To draw conclusions from the cost-effectiveness ratio, it is necessary to compare it with estimates of societal willingness to pay for health gains. If the cost-effectiveness ratio is lower than societal willingness to pay, then it can be considered cost-effective. If the cost-effectiveness ratio is greater than societal willingness to pay, then the programme is considered not cost-effective. A conservative and common threshold of willingness to pay in the USA is $50 000 per QALY saved^(32)^. We conducted a probabilistic sensitivity analysis to generate 95 % CI for the estimates.

*(5) Conduct sensitivity analysis of key assumptions*

We conducted one-way sensitivity analysis to test the sensitivity of results to two key assumptions in the model: (1) programme effectiveness and (2) the ongoing implementation costs to maintain the intervention. Specifically, we examined the change in net costs if effectiveness was reduced by 50 % and if maintenance costs were 50 or 100 % of start-up implementation cost.

## Results

The average total implementation cost of SRCP recipients was $1 264 609, with low variation (sd = $204 819) (Table 4). The most cost-intensive activity for recipients on average was conducting evaluation, but implementing sodium reduction interventions was nearly as costly. There was low variation across all cost categories for recipients with all sd being less than half of the mean. Total monthly costs incurred by SRCP food service organisations averaged $4282 but varied substantially, ranging from $88 to $28 747 (Table 5). Most of this variation was eliminated when considering the cost per person served at the food service organisation. After constructing this measure, only one outlier (more than 3 sd above the mean) remained, and the rest of the values were within a consistent range. This organisation was implementing major recipe changes. The most cost-intensive activity for food service organisations on average was additional food preparation, but only six of the food service organisations reported conducting this activity, and their costs varied widely ($27–$7886). The next most-costly activities were nutritional analysis and recipe development ($1648) and healthy food promotion ($1126), both of which were common activities among partners (conducted by fifteen and twelve partners, respectively).

**Table 4:** Average SRCP recipient implementation cost (30 September 2016–29 September 2019), by activity

Activity	*n*	Mean	sd	Min	Max
Building and maintaining partnerships	8	$201 401	$45 743	$116 265	$263 584
Designing sodium reduction interventions	8	$183 180	$52 091	$109 586	$243 625
Implementing sodium reduction interventions	8	$292 040	$80 905	$198 849	$450 409
Conducting evaluation	8	$311 628	$103 480	$182 895	$533 654
Performing administrative activities	8	$276 359	$88 875	$135 493	$380 532
Total	8	$1 264 609	$204 819	$1 008 240	$1 603 550

**Table 5:** Average monthly implementation cost to food service organisations by activity

Activity	*n*	Mean	sd	Min	Max
Total cost	20	$4282	$6790	$88	$28 747
Total cost per person served	17	$2	$4	$1	$19
Cooking for new product offerings	6	$1964	$2990	$27	$7886
Nutritional analysis and recipe development	15	$1648	$3370	$39	$13 047
Healthy food promotion, including environmental and behavioural economic interventions	12	$1126	$2325	$19	$8062
Other activities related to development	7	$1061	$1326	$15	$3341
Find new lower-sodium ingredients	14	$941	$2342	$11	$8933
Meetings	12	$596	$787	$11	$2399
Other activities	3	$494	$404	$169	$947
Nutrition guideline implementation	13	$432	$952	$5	$3563
Trainings for new recipes or techniques	12	$64	$81	$3	$296

Table 6 shows estimates of the cost of implementation achievements at both the recipient and food service organisation levels. At the recipient level, cost per person reached averaged $10, and cost per food service organisation reached averaged $42 917. Both metrics had moderate variation, with sd near the mean estimates. Median values were $6 and $36 623, respectively. At the food service organisation level, monthly cost per item affected by recipe modification or product substitution averaged $22 869, but this average was driven by one outlier ($183 979); the median was only $684. This outlier was an organisation that had incurred substantial implementation costs but had not yet impacted many menu items.

**Table 6:** Implementation cost-effectiveness measures

Cost-effectiveness metric	*n* *	Mean	sd	Median	Min	Max
Recipient level						
Cost per person reached	8	$10	$10	$6	$1	$28
Cost per food service organisation reached	8	$42 917	$37 343	$36 623	$5308	$122 316
Food service organisation level						
Monthly cost per item affected by recipe modification or product substitution	13	$22 869	$54 290	$684	$3	$183 979

* At the recipient level, N represents the number of recipients that reported both cost data and the performance measure data. At the food services organisation level, N represents the number of food service organisations that reported both cost data and performance measure data.

Table 7 presents estimates of the potential long-term cost-effectiveness of SRCP through 2025 and 2040. If changes made are sustained through 2025, the activities implemented under SRCP are projected to decrease premature deaths by 0·17 % and medical costs by 0·12 % and increase QALY by 0·77 % cumulatively over the entire period among the populations targeted by SRCP recipients. When examining the cost-effectiveness of these impacts, the programme is cost saving, indicating that cumulative medical cost savings through 2025 are greater than cumulative programme costs. If sustained through 2040, the activities implemented under SRCP are projected to decrease deaths by 0·19 % and medical costs by 0·14 % and increase QALY by 0·91 % cumulatively over the entire period among the populations targeted by SRCP recipients. When examining the cost-effectiveness of these impacts, the programme is cost saving, indicating that cumulative medical cost savings through 2040 are greater than cumulative programme costs.

**Table 7:** Long-term cost-effectiveness of SRCP through 2025 and 2040*

	2025		2040	
Metric	Mean	95 % CI	Mean	95 % CI
Percentage change in premature deaths in the target population	−0·17 %	–0·14 %, –0·29 %	−0·19 %	–0·16 %, –0·31 %
Percentage change in average annual QALY per capita in the target population	0·77 %	0·64 %, 1·25 %	0·91 %	0·75 %, 1·45 %
Percentage change in medical costs in the target population	−0·12 %	–0·09 %, –0·21 %	−0·14 %	–0·11 %, –0·24 %
Net cost per capita in the target population (2018 $)	–$1·82	–$1·69, –$1·88	–$2·09	–$1·97, –$2·23
Cost-effectiveness ratio per premature death averted	Cost saving		Cost saving	
Cost-effectiveness ratio per quality-adjusted life-year gained	Cost saving		Cost saving	

* (1) Target population is the total population in the food service organisations targeted by SRCP. (2) Estimates were generated using PRISM’s nationally representative model and measures of SRCP programme implementation and costs. (3) Estimates are for the entire population aged 2+ years.

Table 8 presents one way sensitivity analysis testing the impact of changes in assumptions on the projected impact on per capita net costs through 2025 and 2040. A 50 % reduction in programme effectiveness was estimated to reduce the cost savings of the programme, but net costs were still negative indicating the programme is still projected to be cost saving. After increasing maintenance cost of the programme to 50 and 100 % of start-up implementation cost, the programme is still projected to be cost saving, although the amount saved per capita was reduced.

**Table 8:** One-way sensitivity analysis of change key assumptions on net-cost per capita of SRCP through 2025 and 2040*

Change in assumption	2025	2040
50 % reduction in programme effectiveness	–$0·61	–$0·41
Maintenance costs are 50 % of start-up implementation costs	–$1·27	–$1·24
Maintenance costs are 100 % of start-up implementation costs	–$0·58	–$0·19

* (1) Target population is the total population in the food service organisations targeted by SRCP. (2) Estimates were generated using PRISM’s nationally representative model and measures of SRCP programme implementation and costs. (3) Estimates are for the entire population aged 2+ years.

## Discussion

We evaluated the costs and outcomes of the first 3 years of the 2016–2021 round of SRCP (September 2016–September 2019). We estimated the costs of achieving programme implementation goals and found that at the recipient level, cost per person reached averaged $10, and cost per food service organisation reached averaged $42 917. At the food service organisation level, monthly cost per item affected by recipe modification or product substitution had a median of $684. We also estimated the cost-effectiveness of SRCP through 2025 and 2040. Results demonstrate that SRCP strategies are projected to be cost saving through 2025 and 2040 if the sodium reduction observed in programme performance measures is sustained. The health impacts were not large, but the programme costs are projected to be offset by medical cost savings over time. Furthermore, the health impact could be greater if scaled across a larger population (e.g. the population reached by SRCP was 3·9 million and 20 % reduced sodium consumption). The simulated reduction in sodium intake represented 6 % of the reduction needed to reach the recommended daily intake of 2300 mg^(5)^. This average population level reduction is in line with the average reduction across studies identified in a recent systematic review^(33)^. Understanding the cost of public health programmes and the long-term impacts are key to include in a larger framework for public health decisions and chronic disease prevention^(34)^. The results highlight how sodium reduction strategies can impact health and healthcare cost over time. Programme implementation costs are primarily incurred at the outset, and once changes are in place (e.g. lower-sodium options available), the impact can compound over time with potential cost savings by 2025. However, it is important to note that programme costs are born by public health agencies while the medical cost savings accrue to individuals, payers and health systems.

Previous studies have used simulation models to examine the potential impact of hypothetical changes in sodium intake with no consideration of strategies to achieve the hypothetical changes^(35–38)^. One study examined hypothetical strategies for achieving sodium reduction in the USA, estimating that a government-led collaboration with food manufacturers to reduce sodium content would increase life-years by 1·3 million and save $32·1 billion in lifetime medical costs and a sodium tax would increase life-years by 840 113 and save $22·4 billion in lifetime medical costs^(39)^. However, the assumptions about the efficacy of strategies used in the study are not drawn from practice-based evidence in the USA and implementation costs are not considered. Other studies have simulated the cost-effectiveness of strategies implemented in other countries, such as the United Kingdom and New Zealand, and estimated approaches to be either cost-effective or cost-saving^(40,41)^. The results of this study add to the evidence base by demonstrating what a public health intervention can achieve and incorporating implementation costs for a small set of sodium reduction strategies.

Analysis of the cost of achieving implementation outcomes provides insight into the return on programme inputs, demonstrating how much was achieved for investments, and assists in planning for other organisations seeking to implement similar sodium reduction strategies. There was moderate variation in the recipient cost per person and food service organisation reached, which may be driven by differences in venues that may be more likely to serve more people less frequently (e.g. hospitals) or fewer people more frequently (e.g. congregate meals). This variation is an important consideration for planners seeking to budget sufficiently to achieve programme goals in targeted venues.

Results also highlight the important contributions of partner food service organisations. Past studies have assumed that food service organisations would not incur any additional costs to make recipe modifications or product substitutions because it is part of normal reformulation operations^(39)^. However, the results of this study provide contradictory evidence, demonstrating that food service organisations do incur costs on a range of activities, including recipe modification, procurement changes and overall coordination. This finding is important when considering the feasibility of these sodium reduction strategies because food service organisations may be reluctant to partner given the costs. Other studies of SRCP have shown the importance of external and internal factors to generate buy-in for sodium reduction efforts^(42)^, which are important for overcoming potential cost concerns. Cost per menu item affected was relatively consistent across food service organisations after accounting for one large outlier that had incurred substantial cost but had yet to achieve any impact, indicating that results may be useful across a range of organisations implementing sodium reduction strategies. Partners from seven of the eight venues submitted cost data, but the highest participation rate was from congregate meals and there was not a clear pattern across the other venues. This may impact results if the probability of response was correlated with lower or higher costs.

This study has several limitations. First, we assumed that we are reducing sodium consumption daily in a consistent population, meaning all of the consumers frequenting these food service organisations eat at these venues daily. We tested this assumption in sensitivity analysis where we reduced the effectiveness measure by 50 %. In this analysis, the impact was reduced, but the net effect was still cost saving. Simulations were cost saving up to a 75 % reduction in the effectiveness input. This assumption allows us to estimate a population-level effect. However, we know that at least some of these food service organisations do not have consistent patrons. Similarly, reach is likely not representative of community populations, and we do not have information on how populations at a higher risk for CVD (e.g. those with high blood pressure or other risk factors) were affected. Second, we assume that changes are sustained through 2025 and 2040, which may not be reasonable because organisations might revert to using higher-sodium recipes or products. Third, PRISM uses a nationally representative population to produce estimates of percentage changes in outcomes. The SRCP target population may not reflect this same population mix, which would impact results. Implementation and effectiveness measures reported by recipients were not reported by all organisations for all venues, so we assumed that measures of the percentage of people reducing sodium intake and the average sodium content of foods were generalisable to all organisations and venues. Further, they are only performance measures and may not represent the causal impact of the programme. Fourth, cost data were collected retrospectively and may be subject to recall bias. Fifth, the partner food service organisations that participated in the cost study were a convenience sample, subject to non-response, and our findings may not be generalisable to all SRCP partner food service organisations. Finally, simulation modelling results are limited by the availability and quality of evidence in the literature. PRISM is based on the latest evidence and has been tested and validated extensively but it is subject to these standard model limitations.

By providing evidence of the cost-effectiveness of a public health sodium reduction initiative, this study is an important advance in the literature on the cost-effectiveness of sodium reduction strategies. Community-level nutrition interventions that augment the amounts of micro- and macro-nutrients in foods that people consume without having to change their behaviour have been shown to play a key role in improving population health (e.g. folic acid fortification of foods^(43)^). Community-level change is not easy to achieve, but the success can be substantial and takes the onus off consumers who may not be tracking the nutrient content or who may have constrained options. In food fortification examples, success happened after food fortification policies and standards were set by the US Food and Drug Administration. However, similar to SRCP, food fortification started as a voluntary opt-in by food producers. Sodium reduction strategies at the food service organisation level provide an opportunity to make changes to the amount of a nutrient the population consumes and can affect real health outcomes^(44)^. The findings here represent the current state of implementation but if these efforts could be scaled up, the average daily amount of sodium consumed by US adults could be brought closer to the recommended amount in the dietary guidelines. The results of this study demonstrate the long-term cost-effectiveness of SRCP, which can catalyse future work in sodium reduction and promote scale-up to achieve this impact. Furthermore, the results demonstrate the costs of achieving implementation goals that can support effective planning for future programmes, ensuring that budgets are sufficient to achieve impact.
